# Prognosis and Risk Stratification of Patients with Advanced Heart Failure Followed-Up on an Outpatient Clinic

**DOI:** 10.3390/biomedicines13112743

**Published:** 2025-11-10

**Authors:** Eftychia Papaioannou, Stefania Chatzipanteliadou, Aidonis Rammos, Ilias Gkartzonikas, Aris Bechlioulis, Ilektra Stamou, Vasileios Bouratzis, Lampros Lakkas, Lampros K. Michalis, Katerina K. Naka

**Affiliations:** Second Department of Cardiology, Faculty of Medicine, School of Health Sciences, University Hospital of Ioannina, University of Ioannina, 45110 Ioannina, Greece; efpapaioannou@hotmail.com (E.P.); chatzipanteliadou.s@gmail.com (S.C.); a.rammos@uoi.gr (A.R.); gkartz.ilias@gmail.com (I.G.); md02798@yahoo.gr (A.B.); ilektst@gmail.com (I.S.); v.bouratzis@gmail.com (V.B.); ftpcavalier52@gmail.com (L.L.); lamprosmihalis@uoi.gr (L.K.M.)

**Keywords:** advanced heart failure, levosimendan, intravenous diuresis, mortality risk scores

## Abstract

**Background/Objectives**: Advanced heart failure (AdvHF) characterizes patients with impaired functional capacity, severe systolic or diastolic cardiac function, unplanned visits or hospitalizations, raised natriuretic peptides, and increased mortality. **Methods**: Ninety-five consecutive AdvHF patients followed in a tertiary academic center in Northwestern Greece (2nd Department of Cardiology, University Hospital of Ioannina) were enrolled over a 30-month period. Three distinctive patterns of management were recognized and assessed: intermittent levosimendan administration to 33 patients, intermittent intravenous furosemide administration to 17 patients, and 45 patients were followed up exclusively on an outpatient basis with frequent visits. MAGGIC, SHFM, and BCN-Bio scores were assessed in all patients and mortality was also assessed. **Results**: Mean age was 73 (±10) years, and 38% were females, 41% had diabetes mellitus, 41% had chronic obstructive pulmonary disease, 59% had coronary artery disease (CAD), 73% had a history of atrial fibrillation, and 82.1% had a cardiac device implanted. The median duration of follow-up was 24 months (IQ range 14, 30). The 12-month and 30-month mortality rates were 19% and 49%, respectively. Higher rates of 1-year mortality were observed in the levosimendan group (30%). The median 12-month mortality of the three scores was comparable to the actual mortality, but their prognostic value was not satisfactory (AUC < 0.540 and *p* > 0.05 for all), while they performed better for 30-month mortality (AUC < 0.756 and *p* > 0.05 for all). In the current study, mortality at 12 months was associated with decreasing diastolic blood pressure (DBP) and sodium levels; the presence of CAD (*p* < 0.05 for all) and mortality at 30 months was associated with decreasing systolic blood pressure, as well as DBP and left ventricle ejection fraction, but also with the presence of CAD and the use of renin–angiotensin–aldosterone system blockers. Logistic regression-based models incorporating these factors have a greater diagnostic accuracy (AUC = 0.824 and 0.817 for 12 and 30 months, respectively; *p* < 0.001 for both). **Conclusions**: AdvHF patients represent a complex population requiring close follow-up and novel strategies to improve survival. Larger studies are needed to refine and update predictive scores in this population.

## 1. Introduction

Heart failure (HF) is a clinical syndrome in which the heart’s pumping ability is insufficient to maintain adequate blood flow to the organs, leading to high morbidity and mortality [1,2,3]. The disease severity is classified based on symptom intensity and functional capacity, with the New York Heart Association (NYHA) and American College of Cardiology/American Heart Association (ACC/AHA) classifications most widely used [4]. Advanced HF (AdvHF) characterizes the most challenging, critically ill HF patients. There have been various definitions of AdvHF [5,6], which is typically characterized by a set of clinical signs that indicate that the patient has become unresponsive or resilient to standard treatments, necessitating additional interventions [7].

The primary treatment objectives in AdvHF include hemodynamic stabilization, functional capacity maintenance, symptom alleviation, quality of life preservation, and hospital admission prevention, which is highly associated with mortality [8]. Patients with AdvHF will ultimately either receive a definitive treatment, such as heart transplantation (HTx) or a left ventricular assist device (LVAD) temporarily or permanently, or palliative care (PC) [9].

Towards the stabilization and preparation for further treatment options, AdvHF Clinics are emerging to guide long-term care, with scheduled hospital-based therapies, including levosimendan infusions and proactive intravenous (IV) diuresis, as potential strategies [10,11], while outpatient care also plays a critical role in long-term management. Levosimendan is a calcium-sensitizing agent with inotropic and vasodilatory properties [11] which has shown to improve hemodynamic parameters without increasing myocardial oxygen consumption [12]. Unlike traditional inotropes, which are associated with increased arrhythmic risk and mortality, levosimendan has been suggested as a safer alternative for AdvHF patients, particularly those with recurrent decompensations [13]. Some studies have proposed that intermittent, scheduled administration of levosimendan may reduce hospitalizations and improve functional status [14].

Another important component of AdvHF management is volume regulation, as congestion is the primary cause of HF admissions. Despite the widespread use of oral diuretics, many patients require escalation to IV treatment to maintain euvolemia [15]. Scheduled IV diuresis has emerged as a potential strategy to prevent worsening congestion and reduce hospitalizations by proactively addressing fluid retention [16], though its impact on survival remains uncertain.

Beyond hospital-based IV interventions, optimized outpatient management in experienced focused AdvHF Clinics plays a crucial role in the care of AdvHF patients. Many patients experience a gradual onset and slow progression of signs and symptoms, while others deteriorate rapidly. The potential window, in which effective treatment (e.g., medication titration) potentially halts further deterioration and reduces the likelihood of hospitalization, requires the early identification of those at high risk or with poor prognosis. Several prognostic models have been developed for ambulatory HF patients, with the most common being the Seattle Heart Failure Model (SHFM), the MAGGIC-HF risk score, and the Barcelona Bio-Heart Failure (BCN-Bio-HF) risk calculator [17,18,19]. Each of these models has unique features and limitations that influence their clinical application, but they all depict the severity of the disease and its poor survival rates.

This current retrospective observational study aimed to characterize AdvHF patients attending the outpatient clinic of a tertiary University hospital, with various management strategies and identified factors associated with mortality. The applicability of established prognostic risk scores in the management of these patients was also assessed.

## 2. Materials and Methods

This study was a single-center, retrospective, observational registry that enrolled 95 consecutive patients diagnosed with AdvHF and followed-up in the Heart Failure unit of the Second Department of Cardiology at the University Hospital of Ioannina, Greece, over a 30-month period from January 2023 to June 2025.

All adult patients (*n* = 95) with HF and multiple previous hospitalizations for acute decompensation who were diagnosed with AdvHF based on the ESC criteria [4] were followed-up and studied. Criteria supporting the diagnosis of AdvHF are considered as follows: functional class NYHA III or IV, presence of severe left or right ventricular dysfunction, episodes of pulmonary or systemic congestion requiring high dose IV diuretics, or low cardiac output requiring inotropes or vasoactive drugs, episodes of malignant arrhythmias, or severe impairment of exercise capacity. HF hospitalization refers to an unplanned hospital admission with length of stay more than 24 h due to either worsening signs and symptoms of HF or low cardiac output and arrhythmias, with clinical, laboratory, or invasive signs.

All enrolled patients had given their written informed consent to participate (protocol code: 11855; date of approval: 15 May 2023). In all patients, a comprehensive and meticulous evaluation was conducted, including a detailed medical history, thorough clinical examination, electrocardiogram, echocardiogram, laboratory investigations, and, where applicable, an interrogation of implanted cardiac devices. Patients who had undergone advanced cardiovascular interventions, including heart transplantation or LVAD implantation, or unwilling to give consent, were excluded from the study.

During follow-up, guideline-directed medical therapy (GDMT) for HF management was systematically implemented and optimized, and further interventional strategies regarding myocardial reperfusion (surgical or percutaneous), severe valvular disease management (surgical or percutaneous), and cardiac rhythm device implantation were carefully considered on a case-by-case basis. Specific therapeutic interventions, including intermittent inotropic support and/or intermittent intravenous diuretic therapy, were administered in selected patients with AdvHF according to the physicians’ evaluation of each patient’s profile. Retrospectively, three different management strategies were identified. Intermittent intravenous inotropic support with levosimendan infusion for 8–12 h every 3–4 weeks, along with intravenous diuretics and noradrenaline (depending on the systematic blood pressure levels; systolic blood pressure <90 mmHg), was used in patients with HF with reduced ejection fraction (HFrEF) and low systemic perfusion (Group 1). Intermittent administration of high doses of intravenous furosemide every 2–4 weeks was usually implemented in patients with HF with preserved ejection fraction (HFpEF), or HFrEF without signs of hypoperfusion but persistent congestion and volume overload despite high oral doses of diuretics (Group 2). Frequent follow-up visits in the specialized out-patient HF clinic every 8–10 weeks in more stable but quite symptomatic patients needing relatively lower doses of diuretics (Group 3). In the latter group, in the event of a clinical deterioration, the healthcare team would assess the need for treatment escalation with the administration of either intermittent inotropic support or high doses of intravenous diuresis. Selected patients who were deemed candidates for more advanced HF therapies (i.e., heart transplantation or LVAD) were referred to the national AdvHF center to be screened; only 1 female patient with a history of hypertrophic cardiomyopathy in the burn-out phase who had frequent rehospitalizations for inotrope and diuretics administration underwent heart transplantation and was removed from the final analysis.

For all enrolled subjects, three established prognostic risk scores were calculated based on parameters derived from their first visit to the AdvHF clinic: the SHFM, the MAGGIC score, and the BCN Bio-HF score [17,18,19]. Specifically, the SHFM was utilized to predict 1-year and 5-year survival rates [17], the MAGGIC score was utilized to estimate 1-year and 3-year mortality risk [18], and the BCN Bio-HF score was utilized to assess 1-year and 5-year mortality risk [19].

### Statistical Analysis

Normal distribution of all continuous variables was evaluated using the Kolmogorov–Smirnoff test. Continuous data are presented as mean ± standard deviation or median values (interquartile range), while dichotomous data are presented as number (percentage). Comparisons among the three groups were made using the Fischer x2 test for dichotomous variables and one-way ANOVA or Kruskal–Wallis tests for continuous variables. Univariable logistic regression analysis was used to assess associations of various parameters with mortality. Multivariable logistic regression analysis based on significant univariable associations for mortality (at the level of *p* < 0.05) was used to construct prognostic models. The predictive accuracy of each score for 12- and 30-month all-cause mortality was assessed by the receiver operating characteristic (ROC) curves. The areas under the receiver operating characteristic (ROC) curve (AUCs) with 95% confidence intervals (CIs) were calculated. Comparison of AUCs between different scores and models was performed using the method proposed by Hanley and McNeil [20]. A two-tailed *p*-value < 0.05 was used to determine statistical significance. All analyses were performed with the software IBM SPSS Statistics version 23 (IBM, Armonk, NY, USA).

## 3. Results

A total of 95 HF patients (mean age 73 ± 10 years, 38% females) were available for analysis. Characteristics of the study population are shown in Table 1. The prevalence of diabetes mellitus (DM), established coronary artery disease (CAD), history of atrial fibrillation (AF), and chronic obstructive pulmonary disease (COPD) was 41%, 59%, 73%, and 41%, respectively. Most enrolled patients had a rhythm device; 24% had a simple pacemaker, 11% had a biventricular pacemaker, 23% had a cardioverter–defibrillator, and 24% had a biventricular pacemaker with a defibrillator. The median value of the left ventricular ejection fraction (LVEF) was 30% (IQ range 20, 50). Baseline laboratory findings showed an estimated glomerular filtration rate (eGFR) of 47.6 ± 17.3 mL/min/1.73 m^2^, and median BNP values were 589 pg/mL. Patients were treated with GDMT; β-blockers were used in 73% of patients, sodium-glucose co-transporter 2 inhibitors (SGLT2i) were used in 55% of patients, angiotensin-converting enzyme inhibitors/angiotensin receptor blockers (ACEi/ARBs) or angiotensin receptors/neprilysin inhibitors (ARNIs) were used in 30% of patients, mineralocorticoid receptor antagonists (MRAs) were used in 84% of patients, and hydrochlorothiazide (HCTZ) was used in 11% of patients. The median furosemide dose was 120 mg. The median duration of follow-up was 24 months (IQ range 14, 30) and the median number of prior hospitalizations for AdvHF during the last 12 months was 2 (IQ range 1, 3).

There were 33 patients in the intermittent levosimendan infusion protocol (Group 1), 17 in the intermittent IV furosemide protocol (Group 2), and the other 45 AdvHF patients were followed-up in the Outpatient Clinic without IV interventions (Group 3) (Table 1). Group 2 patients were older, mostly females, had a higher pulse pressure (PP) and LVEF, and a lower prevalence of CAD history and rhythm devices compared to the other patients (*p* < 0.05 for all). Group 2 patients also presented a higher daily furosemide dose compared to the other patients (*p* < 0.001). 

### 3.1. Mortality at 1-Year Follow-Up Analysis

At 12 months, the actual overall mortality was 19%, with higher rates observed in Group 1 (30%) compared to Group 2 (20%) and Group 3 (9%), with a trend toward statistical significance (*p* = 0.059) (Table 2). The median values of the 12-month predicted risk for mortality produced from the three established risk scores were comparable to the actual mortality of the entire population (MAGGIC at 24.8%, SHFM at 16%, and BCN at 16.1% vs. actual 19%) (Table 2).

In the univariable logistic regression analysis evaluating predictors of 1-year mortality in the total population (Table 3), diastolic blood pressure (DBP) (per 5 mmHg increase) (OR 0.61, 95% CI 0.42–0.87; *p* = 0.007), serum sodium (per 1 mmoL/L increase) (OR 0.82, 95% CI 0.68–0.99; *p =* 0.041), and history of established CAD (OR 3.87, 95% CI 1.03–14.59; *p* = 0.046) were associated with 1-year mortality.

**Figure 1:**
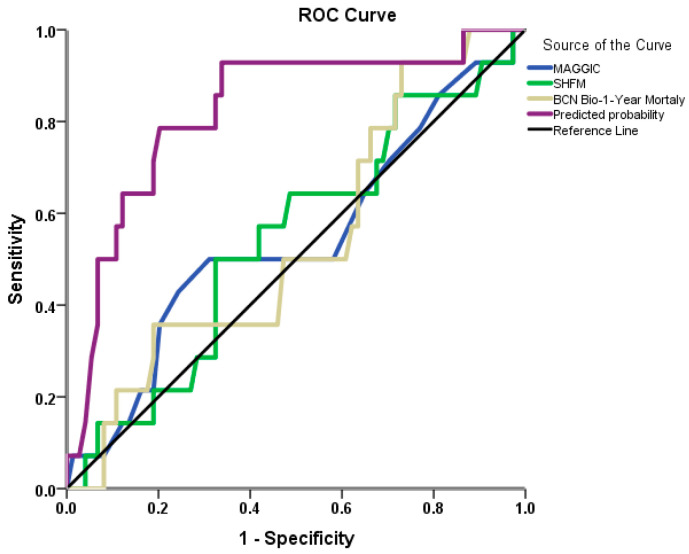
ROC curves for prediction of 1-year mortality with MAGGIC score, SHFM score, and BCN Bio-HF score. The fourth line represents the prediction model produced by the current population, including diastolic blood pressure, sodium, and CAD history.

ROC curve analysis was performed to assess the discriminative performances of established prognostic models for 1-year mortality in our population. The AUC for the MAGGIC score, SHFM, and BCN Bio-HF models were 0.542 (*p* = 0.616), 0.542 (*p* = 0.616), and 0.540 (*p* = 0.636), respectively. The comparison of AUCs of the three risk scores did not reveal any significant difference between these scores (*p* > 0.05 for all comparisons). A multivariable model (Table 4) incorporating DBP, serum sodium, and CAD history showed an AUC of 0.824 (*p* < 0.001) (Figure 1); this was significantly higher compared to any of the other three risk scores (*p* < 0.01 for all comparisons).

Subgroup analysis showed that DBP (OR 0.37 per 5 mmHg increase, 95% CI 0.16–0.82; *p* = 0.014) and sodium levels (OR 0.71 per 1 mmol/l increase, 95% CI 0.5–0.99; *p* = 0.047) were related to mortality in Group 1 patients, while a higher furosemide dose >120 mg od (OR = 12.0, 95% CI 1.10—131.24; *p* = 0.042) was associated with 1-year mortality in Group 3 (Supplementary Table S1).

### 3.2. Mortality at 30-Month Follow-Up Analysis

At 30 months, the actual overall mortality was 49%, with significantly higher mortality rates observed in Group 1 (72%) and Group 2 (71%) patients compared to Group 3 (26%) patients (*p* < 0.001) (Table 5).

In the 30-month analysis, 74 patients that completed the respective follow-up were included. In the univariable logistic regression analysis, systolic blood pressure (SBP) (OR 0.74 per 10 mmHg increase, 95% CI 0.56, 0.96; *p* = 0.025), DBP (OR 0.63 per 5 mmHg increase, 95% CI 0.47, 0.85; *p* = 0.002), LVEF (OR 0.60 per 10% increase, 95% CI 0.43, 0.84; *p* = 0.003), CAD history (OR 3.15, 95% CI 1.15, 8.66; *p* = 0.026), sodium levels (OR 0.81 per 1 mmol/l increase, 95% CI 0.68, 0.96; *p* = 0.015), use of ACEi/ARBs (OR 0.33, 95% CI 0.12, 0.95; *p* = 0.039), use of MRAs (OR 6.07, 95% CI 1.23, 30.03; *p* = 0.027), and higher furosemide daily doses >120 mg (OR 3.02, 95% CI 1.17, 8.03; *p* = 0.022) were associated with mortality (Table 6).

**Figure 2:**
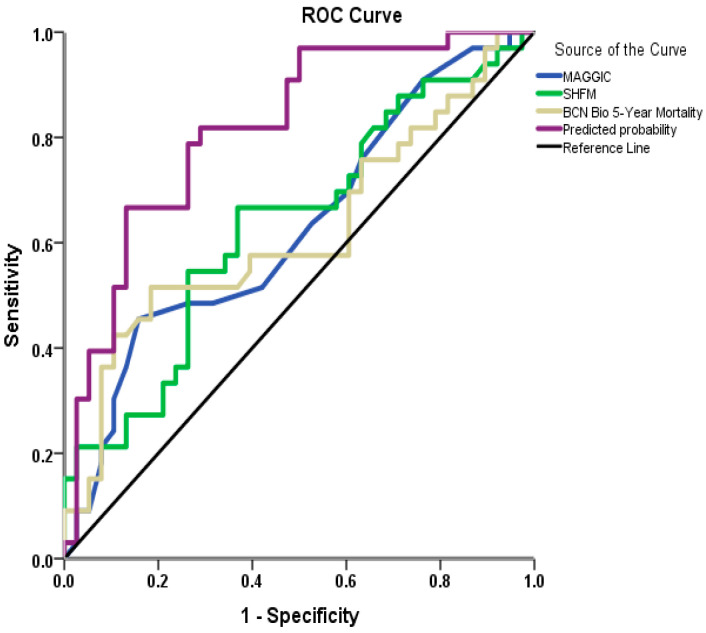
ROC curves for prediction of 30-month mortality with MAGGIC score, SHFM score, and BCN Bio-HF score. The fourth line represents the prediction model produced by the current population, including diastolic blood pressure, sodium, LVEF, and high daily furosemide dose.

In the ROC curve analysis, the AUC values for the three risk models were as follows: MAGGIC at 0.630 (*p* = 0.061), SHFM at 0.636 (*p* = 0.05), and BCN Bio-HF at 0.622 (*p* = 0.078). The comparison of the AUCs of the three risk scores did not reveal any significant difference between these scores (*p* > 0.05 for all comparisons). Based on the univariable analysis, a multivariable logistic regression analysis was performed, and a predictive model that incorporated the independent predictors of 30-month mortality (Table 7) presented a predictive accuracy AUC = 0.817 (*p* < 0.001) (Figure 2). The AUC of the current model was significantly higher compared to MAGGIC (*p* = 0.025), SHFM (*p* = 0.03), and BCN Bio-HF (*p* = 0.022).

In the subgroup analysis, sodium levels (OR 0.61 per 1 mmoL/L increase, 95% CI 0.38, 0.98; *p* = 0.04) and SGLT2i use (OR 11.38, 95% CI 1.17, 110.42; *p* = 0.036) were associated with mortality in Group 1 patients. Furthermore, DBP (OR 0.58 per 5 mmHg increase, 95% CI 0.35, 0.98; *p* = 0.041), LVEF (OR 0.54 per 10% increase, 95% CI 0.32, 0.92; *p* = 0.024), and sodium levels (OR 0.77 per 1 mmoL/L increase, 95% CI) were associated with mortality in Group 3 patients (Supplementary Table S2).

## 4. Discussion

This retrospective observational study provides recent real world data on the characteristics and management of AdvHF patients in a specialized HF unit, which includes an outpatient clinic for the management and long-term follow-up of patients with AdvHF and a day-care unit in which specific interventions are offered to these patients (i.e., periodic intravenous administration of diuretics and inotropes). Currently, there is no availability for advanced HF treatment, such as heart transplantation or ventricular assist devices, and patients eligible for these modalities are usually referred in another institution; only one patient underwent heart transplantation during this time period. Patients with AdvHF are usually enrolled after multiple recent hospitalizations in the department or via referrals from secondary regional hospitals and private sector consulting cardiologists. All therapeutic decisions were made through a collaborative, multidisciplinary approach involving the healthcare team, the patient, and their family, ensuring a patient-based and evidence-based management plan to maximize the clinical benefit. A significant heterogeneity in the phenotype of the study population was observed; during analysis, three main patterns of management strategies were recognized retrospectively with different clinical characteristics, but also with disease progression and prognosis. Smooth transitions across different management strategies were ensured according to the patient’s symptoms, signs of hypoperfusion and congestion, or lack of response to classical guideline-directed medical therapies, including diuretics.

The study population was typical of a general HF population with symptomatic elderly patients (mean age 73 years), including mostly males with a high prevalence of classical comorbidities, which is in agreement with previously reported studies [21]. Severely increased BNP levels along with a high frequency of the use of HF medications, and especially high doses of furosemide (median oral dose of 120 mg), were suggestive for a population with AdvHF. Furthermore, the use of ACE/ATII was relatively low, probably due to intolerability and low BP, which is also suggestive of AdvHF. Interestingly, a large majority had a rhythm device implanted, with approximately 35% receiving resynchronization therapy. Comorbidities were evenly distributed in the various groups studied, except for CAD, which was most commonly encountered in Group 1 patients.

Patients from Group 1, who required the intermittent administration of inotropes (most often levosimendan) plus intravenous diuretics, had HFrEF with severely reduced LVEF (median value 25%), received high doses of daily diuretics (median dose of 160 mg of furosemide), were intolerable to the use of ACEi/ARBS (use in only 9%), and had the greatest prevalence of a defibrillator device (70%) and resynchronization device (46%). These patients had the lowest values of blood pressure and pulse pressure, probably indicating low cardiac output and, in certain cases, hypoperfusion.

Group 2 patients were older (mean age 80 years), mostly females (71%) with HFpEF (median LVEF 50%), with higher blood pressure values and pulse pressure (probably related to increased arterial stiffness), received very higher doses of oral furosemide (median dose of 200 mg), and had the lowest rate of rhythm devices among other groups. These patients were probably characterized by higher diuretic resistance, without signs of hypoperfusion or severely decreased cardiac output, and thus needed frequent intravenous administration of high intravenous furosemide doses.

Finally, the outpatient group (Group 3) consisted of more “stable” but nevertheless highly symptomatic patients with both HFpEF and HFrEF, and also had a high prevalence of rhythm devices and GDMT, with clinical characteristics similar to the other groups but requiring significantly lower doses of diuretics (median furosemide dose of 80 mg).

The intermittent IV inotrope infusion is not a new theory; previous studies with inotropes like milrinone have resulted in safe and efficient hemodynamic and functional improvements in patients with AdvHF [22]. However, there are also controversial data pointing to increased mortality from the use milrinone of dobutamine [23]. Since patients with AdvHF run out of options and are on a trajectory ultimately either to advanced therapies, i.e., heart transplantation or mechanical circulatory support, or to a palliative care pathway [8], other inotropes, like levosimendan, have been tried. Levosimendan’s unique mechanism of action, which enhances myocardial contractility while reducing preload and afterload [24], has been associated with improved exercise tolerance and reduced hospitalizations in previous studies [25]. The first study that compared levosimendan with dobutamine in patients with severe, low-output HF showed lower mortality for up to 180 days [26], but the long-term survival benefits remained uncertain in patients with cardiogenic shock and low cardiac output syndrome [27]. There are reports that levosimendan resulted in combined HF admissions, unplanned HF visits, and death reduction (56.3% vs. 81.4%; *p* < 0.0001) during the first year, but no exclusive mortality reduction [28]. These findings are in line with the evidence suggesting that intermittent levosimendan infusions may serve as a valuable adjunctive therapy for selected AdvHF patients, since none of the Group 1 patients had further HF hospitalizations, but mortality in this group remained high.

Many studies have shown that intravenous diuretic treatment in HF populations has improved the functional capacity of the patients, increased weight loss [29], alleviated symptoms like dyspnea, as well as prevented congestion-related complications, and reduced HF hospitalizations [30] and health costs [31]. However, none of them managed to show a reduction in mortality. In this study, risk prediction in the furosemide group was unreliable, perhaps underscoring the limitations of applying traditional risk models in very small samples.

In accordance with the wide phenotype heterogeneity among the three groups, mortality occurrence was also different in the three groups for either short-term (12-month) or medium-term (30-month), but remained high in both occasions (19% and 49%, respectively). There are numerous risk scores for short- and long-term mortality prediction [32,33], with more [34,35,36] or less encouraging results [37]. Three commonly used HF risk models were currently chosen—MAGGIC, SHFM, and BCN Bio—on the grounds of clinical applicability, and used more than one to minimize the individual inherent weaknesses. Several previous studies have compared multiple risk scores that estimate outcomes in HF patients to demonstrate superiority over the others. A recent study [38] compared four risk scores, without demonstrating clear superiority among them. It was suggested that the choice of risk score to be used should consider the clinical context, availability of data, and research objectives of risk stratification. Seven models that predict inpatient mortality in hospitalized patients had a similar performance, with c-statistics between 0.7 and 0.8; the decision concerning which tool should be used depends on the practical concerns and intended use [39].

The observed mortality in the current studied population was high, as reported in the bibliography [40]. The performance of risk scores in predicting 1-year mortality was very bad, since all three scores had a very low predictive accuracy for outcomes in this population (AUC close to 0.5), although the median numerical score predicted risk for mortality was quite close to the observed score (no significant difference). On the other hand, while all risk scores had a moderate predictive accuracy (AUC 0.600–0.650) for 30-month mortality, the median numerical predicted risk score for mortality was higher than the observed score. It should be considered that, in at least two of the scores, SHFM and BCN Bio-HF, the prediction stands for 5 years instead for 30 months, while the MAGGIC score predicts 3-year mortality. Overestimation of mortality for the whole population likely reflects the evolution in management of HF patients, reflecting the optimization of medication and geographical differences in HF outcomes [41,42]. Therefore, the current study might highlight the need for the renewal of predictive scores with contemporary, more precise prognostic tools [43]. Patients with advanced heart failure (AdvHF) over 80 years old, who are underrepresented in most clinical studies, were also included. On the one hand, traditional HF management relies on pharmacologic interventions, along with implantable devices or transcatheter valve interventions [16]; on the other hand, the clinical benefit of GDMT and adherence to the guidelines is not so robust for octogenarians with HFrEF in terms of short- and long term mortality or 90-day HF readmissions [44]. Moreover, the performance of risk scores cannot be expected to be equally efficacious in various studied groups/phenotypes, and further refinement in risk prediction will be needed. Group 1 had the highest 1-year mortality (*p* = 0.059), whereas Groups 2 and 3 appeared to have better outcomes, although their small sample size limits definitive conclusions. For the 30-month period, Group 3 patients had significantly lower mortality (*p* < 0.001) compared to the other two groups.

Prognostic models for 12- and 30-month mortality produced by the current dataset revealed other significant variables (compared to the ones used in the risk scores) with the ability to perform better than the established risk scores. Although these findings might be an overfitting bias, hemodynamic factors, congestion-related factors, cardiac function indices, and other clinical parameters (i.e., CAD history) should be taken into account, and may be more appropriate for risk prediction in the specific population in future studies. Low DBP may be a marker of either low cardiac output or arterial stiffening, and it has been associated with HF hospitalizations and increased cardiovascular mortality [45]. In HF, low sodium levels and hyponatremia are the results of either volume overload (activation of the sympathetic nervous system leading to peripheral vasoconstriction, sodium, and water retention) or excessive use of natriuretics [46]. In any case, low sodium has been associated with poor outcomes in patients with HF [47]. In the present study, low serum sodium was significantly associated with increased 12-month mortality in Group 1, and 30-month mortality for the whole population and Group 3 separately. Moreover, high diuretic dose, ACEi/ARB use (patients who tolerate this are probably better), CAD, and low LVEF should also be considered. These findings are in accordance with the three established risk scores used, since most of the statistically significant variables are incorporated in them. For instance, the MAGGIC score uses LVEF and the use of ACEi/ARBS; the Seattle HF model incorporates LVEF, ACEi/ARBs, diuretics, and sodium levels, while the BCN Bio-HF score includes sodium levels, LVEF, diuretic dosage, and ACEi/ARB use [17,18,19].

The results of this study contribute to the ongoing discourse on the optimal management of AdvHF, highlighting the potential benefits of structured hospital-based but community-oriented interventions and comprehensive outpatient care, which is a cornerstone of AdvHF care [14,48]. Implementing such programs on a large scale remains challenging due to resource constraints and variability in healthcare infrastructure. In Greece, the unmet need of developing a national HF clinic network has been addressed [49]; this could guide patients that fulfill the criteria in a specialized clinic earlier, before they reach the very advanced or end stages of the disease, when they are not eligible to receive more advanced therapies. The results also suggest that proactive treatment strategies may play a critical role in stabilizing patients, preventing acute decompensations and, ultimately, improving survival outcomes, like in Group 3. Identifying the most suitable strategy for each patient requires careful consideration of disease severity, comorbidities, and response to therapy [48]. Future research should focus on the development and validation of contemporary HF and AdvHF risk models based on specific clinical settings and patient characteristics. Inclusion of biomarkers, patient-reported outcomes, frailty indices, and dynamic clinical variables may enhance the accuracy and relevance of prediction tools. Randomized trials evaluating the role of IV therapies, such as levosimendan and diuretics, are also warranted so to clarify their impact on survival and quality of life. Finally, multidisciplinary HF programs, which integrate cardiologists, nurses, pharmacists, and dietitians, and which have been shown to enhance the adherence to GDMT and facilitate early intervention for decompensations [50], should be strategically implemented in all HF clinics. The results of the current study should be interpreted with caution in any case, and considered as exploratory and hypothesis-generating for future larger studies in populations with unmet needs (i.e., AdvHF patients) in terms of risk prediction and proper management.

## 5. Limitations

This study provides valuable insights into the profile and management of AdvHF; however, several limitations that may affect the interpretation and broad applicability of the findings must be acknowledged. First, this was a single-center study with a limited sample size, which inherently restricts the generalizability of the results to other healthcare settings or populations, as variations in patient demographics, institutional resources, and treatment protocols may influence the outcomes. The lack of a fully organized network for AdvHF that could guide patients meeting the criteria in a specialized clinic earlier is an unmet need. In Greece, there is limited access to advanced HF therapies, such as ventricular assist devices and heart transplantation, and this may differentiate local management strategies compared to other settings and other countries.

The population included in the analysis is rather heterogeneous in terms of patient gender, age, socioeconomic status, and other factors. Overfitting is probably inevitable in studies of a small sample size like the current one; hence, the results of the current study should be interpreted as preliminary and exploratory, and larger studies will be needed to confirm the validity of our observations. Subgroup analyses may be affected by the small sample size to a greater degree, and these results should be interpreted with further caution. Data for medications at the end of the follow-up are not presented. Several up- or down-titration efforts of medications by the physicians may have been made during the follow-up depending on the patients’ symptoms. The relatively short follow-up period limits the ability to assess the long-term effects of the interventions, particularly with respect to critical outcomes such as mortality and disease progression. The cause of mortality was not identified properly in all patients, and thus a relevant analysis was not performed.

## 6. Conclusions

In this retrospective study of AdvHF patients, distinct management strategies were observed that were fit to different patient profiles according to physician decisions. Nevertheless, the short- and medium-term mortality was quite high, with significant intergroup variability, suggesting that specific treatment targets should be set. These findings highlight the importance of individualized monitoring and suggest that other parameters may be of greater importance in predicting survival in AdvHF patients. Currently used risk scores do not seem to perform well in this population, and new parameters must likely be implemented in the management of these patients.

## Figures and Tables

**Table 1:** Baseline characteristics and comparisons among study groups.

	Total Population (*n* = 95)	Group 1 (*n* = 33)	Group 2 (*n* = 17)	Group 3 (*n* = 45)	*p*-Value
Age (years)	73 ± 10	72 ± 10 *	80 ± 7	72 ± 10 *	0.017
Female gender, *n* (%)	33 (38)	6(18)	12 (71)	15 (33)	0.001
Body mass index (kg/m^2^)	26.9 ± 5.7	26.2 ± 4.6	27.2 ± 5.9	27.3 ± 6.3	0.707
Systolic BP (mmHg)	110 ± 18	105 ± 15	118 ± 18	111 ± 20	0.062
Diastolic BP (mmHg)	68 ± 11	66 ± 11	67 ± 10	70 ± 10	0.179
Pulse pressure (mmHg)	42 ± 14	39 ± 10 *	51 ± 16	41 ± 14 *	0.007
LVEF (%)	30 (20, 50)	25 (20, 30)	50 (30, 55)	40 (20, 55)	<0.001
Smoking, *n* (%)	15 (16)	8 (24)	0 (0)	7 (16)	0.084
Hypertension, *n* (%)	57 (60)	21 (64)	10 (59)	26 (58)	0.868
Diabetes, *n* (%)	39 (41)	13 (39)	6 (35)	20 (44)	0.785
CAD, *n* (%)	56 (59)	25 (76)	5 (29)	26 (58)	0.007
COPD, *n* (%)	39 (41)	12 (36)	6 (35)	21 (47)	0.572
Atrial fibrillation, *n* (%)	70 (73)	23 (70)	14 (82)	33 (73)	0.627
Hemoglobin (g/dL)	12.6 ± 2.0	13.1 ± 2.1	12.2 ± 2.1	12.4 ± 2.0	0.200
Na+ (mmoL/L)	137 ± 3	136 ± 3	138 ± 2	137 ± 3	0.268
K+ (mmoL/L)	4.22 ± 0.50	4.02 ± 0.44 **	4.00 ± 0.46 **	4.44 ± 0.48	<0.001
eGFR (ml/min/1.73 m^2^)	47.6 ± 17.3	46.8 ± 18.1	49.1 ± 18.4	47.6 ± 16.7	0.909
Troponin I (ng/mL)	25.0 (14.2, 43.1)	28.0 (16.8, 47.1)	18.8 (14.3, 36.6)	22.4 (11.9, 44.7)	0.275
BNP (pg/mL)	589 (363, 897)	626 (404, 907)	425 (283, 769)	645 (371, 1047)	0.067
Medications, *n* (%)					
B-blockers	69 (73)	25 (76)	10 (59)	34 (76)	0.370
SGLT2I	52 (55)	17 (52)	10 (59)	25 (56)	0.876
ACEi/ARB	28 (30)	3 (9)	7 (41)	18 (40)	0.006
MRA	80 (84)	32 (97)	14 (82)	34 (76)	0.037
Hydrochlorothiazide	10 (11)	4 (12)	3 (18)	3 (7)	0.424
Furosemide dose (mg)	120 (80, 240)	160 (110, 250)	200 (120, 250)	80 (40, 120)	<0.001
Rhythm devices, *n* (%)					
PCM	23 (24)	3 (9)	5 (29)	15 (33)	
CRTP	10 (11)	0 (0)	0 (0)	10 (22)	
ICD	22 (23)	8 (24)	1 (6)	13 (29)	
CRTD	23 (24)	15 (46)	1 (6)	7 (16)	<0.001

* *p* < 0.05 post hoc comparison vs. Group 2; ** *p* < 0.05 post hoc comparison vs. Group 3. Continuous data are presented as mean ± standard deviation or median values (IQR). Dichotomous data are presented as number (percentage). Abbreviations: ACEi/ARB = angiotensin-converting enzyme inhibitor/angiotensin II receptor blocker; BNP = brain natriuretic peptide; BP = blood pressure; CAD = coronary artery disease; COPD = chronic obstructive pulmonary disease; CRTD = cardiac resynchronization therapy defibrillator; CRTP = cardiac resynchronization therapy pacemaker; eGFR = estimated glomerular filtration rate; ICD = implantable cardioverter defibrillator; LVEF = left ventricle ejection fraction; MRA = mineralocorticoid receptor antagonist; PCM = pacemaker; SGLT2I = sodium-glucose co-transporter 2 inhibitor.

**Table 2:** Actual and predicted mortality for patients completing 12 months of follow-up.

	Total Population (*n* = 92)	Group 1 (*n* = 33)	Group 2 (*n* = 15)	Group 3 (*n* = 44)	*p*-Value
Actual mortality	17 (19)	10 (30)	3 (20)	4 (9)	0.059
MAGGIC score	30 (27, 35)	34 (27, 38)	29 (27, 32)	30 (26, 34)	0.139
MAGGIC 1-year mortality	24.8 (19.1, 36.9)	34.2 (19.1, 45.8)	22.7 (19.1, 29.2)	24.8 (17.5, 34.2)	0.139
SHFM score	1.46 (0.98, 2.22)	1.52 (1.14, 2.25)	1.82 (0.99, 2.65)	1.38 (0.80, 2.12)	0.197
SHFM 1-year survival	84 (69, 90)	83 (68, 88)	78 (56, 90)	85 (71, 91)	0.203
BCN 1-year mortality	16.1 (8.2, 28.4)	16.1 (8.5, 30.2)	25.9 (16.0, 31.3)	15.3 (6.2, 26.7)	0.174

BCN Bio = Barcelona bio-heart failure risk calculator; MAGGIC = Meta-Analysis Global Group in Chronic Heart Failure Risk Score; SHFM = Seattle Heart Failure Model.

**Table 3:** Univariable logistic regression analysis for 1-year mortality in the total population.

	Odds Ratio	95% Confidence Interval	*p*-Value
Age/10-year increase	1.30	0.74, 2.29	0.368
Female	0.34	0.09, 1.29	0.112
BMI per 5 kg/m^2^ increase	0.78	0.46, 1.31	0.343
SBP per 10 mmHg increase	0.81	0.58, 1.12	0.204
DBP per 5 mmHg increase	0.61	0.42, 0.87	0.007
PP per 10 mmHg increase	1.07	0.74, 1.57	0.710
LVEF per 10% increase	0.77	0.53, 1.11	0.163
Smoking	2.71	0.79, 9.34	0.115
Hypertension	0.67	0.23, 1.94	0.460
Diabetes	0.50	0.16, 1.57	0.236
Coronary artery disease	3.87	1.03, 14.59	0.046
COPD	1.78	0.62, 5.14	0.284
Atrial fibrillation	1.82	0.47, 6.97	0.385
Hemoglobin per 1 g/dL increase	1.00	0.77, 1.31	0.974
Na^+^ per 1 mmoL/L increase	0.82	0.68, 0.99	0.041
K^+^ per 0.5 mmoL/L increase	0.60	0.33, 1.08	0.09
eGFR per 10 mL/min increase	1.09	0.81, 1.47	0.555
Troponin I > 25 ng/mL	1.22	0.42, 3.52	0.711
BNP > 600 pg/mL	0.99	0.35, 2.88	0.995
Medications			
B-blockers	1.26	0.37, 4.32	0.709
SGLT2i	1.60	0.54, 4.79	0.397
ACEi/ARB	0.46	0.12, 1.74	0.249
MRA	3.67	0.45, 30.05	0.225
Hydrochlorothiazide	0.52	0.06, 4.49	0.555
Furosemide dose > 120 mg	1.43	0.50, 4.12	0.505

ACEi/ARB = angiotensin-converting enzyme inhibitor/angiotensin II receptor blocker; BMI = body mass index; BNP = brain natriuretic peptide; COPD = chronic obstructive pulmonary disease; DBP = diastolic blood pressure; eGFR = estimated glomerular filtration rate; LVEF = left ventricle ejection fraction; MRA = mineralocorticoid receptor antagonist; PP = pulse pressure; SBP = systolic blood pressure; SGLT2I = sodium-glucose co-transporter 2 inhibitor.

**Table 4:** Independent predictors of 12-month mortality in multivariable logistic regression analysis in the total population.

	Odds Ratio	95% Confidence Interval	*p*-Value
Diastolic blood pressure per 5 mmHg increase	0.60	0.40, 0.91	0.015
Coronary artery disease	3.89	1.01, 16.95	0.05
Na^+^ per 1 mmoL/L increase	0.77	0.61, 0.97	0.024

**Table 5:** Actual and predicted mortality for patients completing 30 months of follow-up.

	Total Population (*n* = 74)	Group 1 (*n* = 29)	Group 2 (*n* = 7)	Group 3 (*n* = 38)	*p*-Value
Actual mortality	36 (49)	21 (72)	5 (71)	10 (26)	<0.001
MAGGIC	30 (27, 36)	34 (27, 38)	29 (27, 32)	30 (26, 34)	0.142
MAGGIC 3-year mortality	52.3 (42.7, 70.1)	65.8 (42.7, 78,7)	49.0 (42.7, 59.0)	52.3 (39.7, 65.8)	0.142
SHFM	1.46 (1.05, 2.11)	1.52 (1.14, 2.07)	2.06 (0.61, 2.44)	1.39 (0.81, 2.16)	0.591
SHFM 5-year survival	42 (19, 56)	40 (20, 54)	21 (10, 67)	44 (18, 64)	0.617
BCN 5-year mortality	67.5 (42.8, 91.2)	67.6 (48.2, 89.9)	85.6 (71.1, 91.1)	65.8 (36.8, 92.3)	0.619

BCN = Barcelona bio-heart failure risk calculator; MAGGIC = Meta-Analysis Global Group in Chronic Heart Failure Risk Score; SHFM = Seattle Heart Failure Model.

**Table 6:** Univariable analysis for mortality at 30 months (total population, *n* = 74).

	Odds Ratio	95% Confidence Interval	*p*-Value
Age per 10-year increase	1.23	0.77, 1.97	0.394
Female	0.49	0.18, 1.37	0.173
BMI per 5 kg/m^2^ increase	0.95	0.64, 1.40	0.794
SBP per 10 mmHg increase	0.74	0.56, 0.96	0.025
DBP per 5 mmHg increase	0.63	0.47, 0.85	0.002
PP per 10 mmHg increase	0.87	0.63, 1.22	0.429
LVEF per 10% increase	0.60	0.43, 0.84	0.003
Smoking	0.91	0.29, 2.82	0.863
Hypertension	1.30	0.50, 3.37	0.584
Diabetes	1.37	0.55, 3.45	0.502
Coronary artery disease	3.15	1.15, 8.66	0.026
COPD	1.10	0.44, 2.76	0.839
Atrial fibrillation	1.91	0.66, 5.59	0.236
Hemoglobin per 1 g/dL increase	0.97	0.77, 1.22	0.817
Na per 1 mmoL/L increase	0.81	0.68, 0.96	0.015
K per 0.5 mmoL/L increase	0.66	0.41, 1.09	0.100
eGFR per 10 mL/min increase	0.99	0.76, 1.30	0.944
Troponin I > 25 ng/mL	1.47	0.59, 3.70	0.412
BNP > 600 pg/mL	1.39	0.55, 3.52	0.487
Medications			
B-blockers	0.93	0.32, 2.69	0.895
SGLT2i	2.41	0.95, 6.13	0.065
ACEi/ARB	0.33	0.12, 0.95	0.039
MRA	6.07	1.23, 30.03	0.027
Hydrochlorothiazide	1.64	0.26, 10.41	0.602
Furosemide dose > 120 mg	3.02	1.17, 8.02	0.022

ACEi/ARB = angiotensin-converting enzyme inhibitor/angiotensin II receptor blocker; BMI = body mass index; BNP = brain natriuretic peptide; COPD = chronic obstructive pulmonary disease; DBP = diastolic blood pressure; eGFR = estimated glomerular filtration rate; LVEF = left ventricle ejection fraction; MRA = mineralocorticoid receptor antagonist; PP = pulse pressure; SBP = systolic blood pressure; SGLT2I = sodium-glucose co-transporter 2 inhibitor.

**Table 7:** Independent predictors of 30-month mortality in multivariable logistic regression analysis in the total population (*n* = 74).

	Odds Ratio	95% Confidence Interval	*p*-Value
Diastolic blood pressure per 5 mmHg increase	0.68	0.49, 0.94	0.018
Left ventricular ejection fraction per 10% increase	0.69	0.48, 0.99	0.042
Na per 1 mmoL/L increase	0.84	0.71, 1.00	0.05

## Data Availability

The data presented in this study are available on request from the corresponding author. The data are not publicly available due to privacy issues.

## Supplementary Materials

The following supporting information can be downloaded at: https://www.mdpi.com/article/10.3390/biomedicines13112743/s1, Table S1: Subgroup analysis for factors affecting 1-year mortality; Table S2: Subgroup analysis for factors affecting 30-month mortality.

## Abbreviations

The following abbreviations are used in this manuscript:

ACC	American College of Cardiology
ACE	Angiotensin-converting enzyme inhibitor
AdvHF	Advanced heart failure
AHA	American Heart Association
ARB	Angiotensin II receptor blocker
AF	Atrial fibrillation
BB	Beta blocker
BCN Bio	Barcelona bio-heart failure risk calculator
BMI	Body mass index
BNP	Brain natriuretic peptide
CAD	Coronary artery disease
COPD	Chronic obstructive pulmonary disease
CRTD	Cardiac resynchronization therapy defibrillator
CRTP	Cardiac resynchronization therapy pacemaker
DBP	Diastolic blood pressure
DM	Diabetes mellitus
eGFR	Estimated glomerular filtration rate
GDMT	Guideline-directed medical therapy
HB	Hemoglobin
HCT	Hydrochlorothiazide
HF	Heart failure
HFpEF	Heart failure with preserved ejection fraction
HFrEF	Heart failure with reduced ejection fraction
HTx	Heart transplantation
ICD	Implantable cardioverter defibrillator
IV	Intravenous
LVAD	Left ventricular assist device
LVEF	Left ventricle ejection fraction
MAGGIC	Meta-Analysis Global Group in Chronic Heart Failure Risk Score
MRA	Mineralocorticoid receptor antagonist
NYHA	New York Heart Association
PC	Palliative care
PCM	Pacemaker
PP	Pulse pressure
SBP	Systolic blood pressure
SGLT2I	Sodium-glucose co-transporter 2 inhibitor
SHFM	Seattle Heart Failure Model
TNI	Troponin-I

## References

[1] Ponikowski P., Anker S.D., AlHabib K.F., Cowie M.R., Force T.L., Hu S., Jaarsma T., Krum H., Rastogi V., Rohde L.E. (2014). Heart failure: Preventing disease and death worldwide. ESC Heart Fail..

[2] Schmidt M., Ulrichsen S.P., Pedersen L., Bøtker H.E., Sørensen H.T. (2016). Thirty-year trends in heart failure hospitalization and mortality rates and the prognostic impact of comorbidity: A Danish nationwide cohort study. Eur. J. Heart Fail..

[3] Severino P., Mather P.J., Pucci M., D’Amato A., Mariani M.V., Infusino F., Birtolo L.I., Maestrini V., Mancone M., Fedele F. (2019). Advanced Heart Failure and End-Stage Heart Failure: Does a Difference Exist. Diagnostics.

[4] Crespo-Leiro M.G., Metra M., Lund L.H., Milicic D., Costanzo M.R., Filippatos G., Gustafsson F., Tsui S., Barge-Caballero E., De Jonge N. (2018). Advanced heart failure: A position statement of the Heart Failure Association of the European Society of Cardiology. Eur. J. Heart Fail..

[5] Peled Y., Ducharme A., Kittleson M., Bansal N., Stehlik J., Amdani S., Saeed D., Cheng R., Clarke B., Dobbels F. (2024). International Society for Heart and Lung Transplantation Guidelines for the Evaluation and Care of Cardiac Transplant Candidates-2024. J. Heart Lung Transpl..

[6] Garascia A., Palazzini M., Tedeschi A., Sacco A., Oliva F., Gentile P. (2023). Advanced heart failure: From definitions to therapeutic options. Eur. Heart J. Suppl. J. Eur. Soc. Cardiol..

[7] Strangl F., Ullrich A., Oechsle K., Bokemeyer C., Blankenberg S., Knappe D., Reichenspurner H., Bernhardt A.M., Barten M.J., Rybczynski M. (2020). Assessing palliative care need in left ventricular assist device patients and heart transplant recipients. Interact. Cardiovasc. Thorac. Surg..

[8] Conti N., Gatti M., Raschi E., Diemberger I., Potena L. (2021). Evidence and Current Use of Levosimendan in the Treatment of Heart Failure: Filling the Gap. Drug Des. Dev. Ther..

[9] Setoguchi S., Stevenson L.W., Schneeweiss S. (2007). Repeated hospitalizations predict mortality in the community population with heart failure. Am. Heart J..

[10] Jiang G.Y., Lee C., Kearing S.A., Wadhera R.K., Gavin M.C., Wasfy J.H., Zeitler E.P. (2024). IV Diuresis in Alternative Treatment Settings for the Management of Heart Failure: Implications for Mortality, Hospitalizations and Cost. J. Card. Fail..

[11] Papp Z., Csapó K., Pollesello P., Haikala H., Edes I. (2005). Pharmacological mechanisms contributing to the clinical efficacy of levosimendan. Cardiovasc. Drug Rev..

[12] Silva-Cardoso J., Ferreira J., Oliveira-Soares A., Martins-de-Campos J., Fonseca C., Lousada N., Ilídio-Moreira J., Rabaçal C., Damasceno A., Amorim S. (2009). Effectiveness and safety of levosimendan in clinical practice. Rev. Port. Cardiol..

[13] Papp Z., Agostoni P., Alvarez J., Bettex D., Bouchez S., Brito D., Černý V., Comin-Colet J., Crespo-Leiro M.G., Delgado J.F. (2020). Levosimendan Efficacy and Safety: 20 years of SIMDAX in Clinical Use. Card. Fail. Rev..

[14] ter Maaten J.M., Valente M.A., Damman K., Hillege H.L., Navis G., Voors A.A. (2015). Diuretic response in acute heart failure-pathophysiology, evaluation, and therapy. Nature reviews. Cardiology.

[15] Banerjee P., Tanner G., Williams L. (2012). Intravenous diuretic day-care treatment for patients with heart failure. Clin. Med..

[16] McDonagh T.A., Metra M., Adamo M., Gardner R.S., Baumbach A., Böhm M., Burri H., Butler J., Čelutkienė J., Chioncel O. (2021). 2021 ESC Guidelines for the diagnosis and treatment of acute and chronic heart failure. Eur. Heart J..

[17] Levy W.C., Mozaffarian D., Linker D.T., Sutradhar S.C., Anker S.D., Cropp A.B., Anand I., Maggioni A., Burton P., Sullivan M.D. (2006). The Seattle Heart Failure Model: Prediction of survival in heart failure. Circulation.

[18] Pocock S.J., Ariti C.A., McMurray J.J., Maggioni A., Køber L., Squire I.B., Swedberg K., Dobson J., Poppe K.K., Whalley G.A. (2013). Predicting survival in heart failure: A risk score based on 39,372 patients from 30 studies. Eur. Heart J..

[19] Lupón J., de Antonio M., Vila J., Peñafiel J., Galán A., Zamora E., Urrutia A., Bayes-Genis A. (2014). Development of a novel heart failure risk tool: The barcelona bio-heart failure risk calculator (BCN bio-HF calculator). PLoS ONE.

[20] Hanley J.A., McNeil B.J. (1983). A method of comparing the areas under receiver operating characteristic curves derived from the same cases. Radiology.

[21] Dunlay S.M., Roger V.L., Killian J.M., Weston S.A., Schulte P.J., Subramaniam A.V., Blecker S.B., Redfield M.M. (2021). Advanced Heart Failure Epidemiology and Outcomes: A Population-Based Study. JACC Heart Fail..

[22] Cesario D., Clark J., Maisel A. (1998). Beneficial effects of intermittent home administration of the inotrope/vasodilator milrinone in patients with end-stage congestive heart failure: A preliminary study. Am. Heart J..

[23] Levine B.S. (2000). Intermittent positive inotrope infusion in the management of end-stage, low-output heart failure. J. Cardiovasc. Nurs..

[24] Nieminen M.S., Fruhwald S., Heunks L.M., Suominen P.K., Gordon A.C., Kivikko M., Pollesello P. (2013). Levosimendan: Current data, clinical use and future development. Heart Lung Vessel..

[25] Comín-Colet J., Manito N., Segovia-Cubero J., Delgado J.F., García-Pinilla J.M., Almenar L., Crespo-Leiro M.G., Sionis A., Blasco T., Pascual-Figal D. (2018). Efficacy and safety of intermittent intravenous outpatient administration of levosimendan in patients with advanced heart failure: The LION-HEART multicentre randomized trial. Eur. J. Heart Fail..

[26] Follath F., Cleland J.G., Just H., Papp J.G., Scholz H., Peuhkurinen K., Harjola V.P., Mitrovic V., Abdalla M., Sandell E.P. (2002). Efficacy and safety of intravenous levosimendan compared with dobutamine in severe low-output heart failure (the LIDO study): A randomised double-blind trial. Lancet.

[27] Schumann J., Henrich E.C., Strobl H., Prondzinsky R., Weiche S., Thiele H., Werdan K., Frantz S., Unverzagt S. (2018). Inotropic agents and vasodilator strategies for the treatment of cardiogenic shock or low cardiac output syndrome. Cochrane Database Syst. Rev..

[28] Dobarro D., Donoso-Trenado V., Solé-González E., Moliner-Abós C., Garcia-Pinilla J.M., Lopez-Fernandez S., Ruiz-Bustillo S., Diez-Lopez C., Castrodeza J., Méndez-Fernández A.B. (2023). Intermittent inotropic support with levosimendan in advanced heart failure as destination therapy: The LEVO-D registry. ESC Heart Fail..

[29] Buckley L.F., Carter D.M., Matta L., Cheng J.W., Stevens C., Belenkiy R.M., Burpee L.J., Young M.A., Weiffenbach C.S., Smallwood J.A. (2016). Intravenous Diuretic Therapy for the Management of Heart Failure and Volume Overload in a Multidisciplinary Outpatient Unit. JACC Heart Fail..

[30] Ryder M., Murphy N.F., McCaffrey D., O’Loughlin C., Ledwidge M., McDonald K. (2008). Outpatient intravenous diuretic therapy; potential for marked reduction in hospitalisations for acute decompensated heart failure. Eur. J. Heart Fail..

[31] Verma V., Zhang M., Bell M., Tarolli K., Donalson E., Vaughn J., Hickey G.W. (2021). Outpatient Intravenous Diuretic Clinic: An Effective Strategy for Management of Volume Overload and Reducing Immediate Hospital Admissions. J. Clin. Med. Res..

[32] Luo Y., Li Z., Liu J., Chong Y., Wu B. (2021). Prognostic value of the albumin-bilirubin score in critically ill patients with heart failure. Ann. Palliat. Med..

[33] Scrutinio D., Guida P., Ammirati E., Oliva F., Passantino A. (2025). Risk scores did not reliably predict individual risk of mortality for patients with decompensated heart failure. J. Clin. Epidemiol..

[34] Averbuch T., Zafari A., Islam S., Lee S.F., Sankaranarayanan R., Greene S.J., Mamas M.A., Pandey A., Van Spall H.G. (2025). Comparative performance of risk prediction indices for mortality or readmission following heart failure hospitalization. ESC Heart Fail..

[35] Blumer V., Mentz R.J., Sun J.L., Butler J., Metra M., Voors A.A., Hernandez A.F., O’Connor C.M., Greene S.J. (2021). Prognostic Role of Prior Heart Failure Hospitalization Among Patients Hospitalized for Worsening Chronic Heart Failure. Circ. Heart Fail..

[36] Iliadis C., Spieker M., Kavsur R., Metze C., Hellmich M., Horn P., Westenfeld R., Tiyerili V., Becher M.U., Kelm M. (2021). “Get with the Guidelines Heart Failure Risk Score” for mortality prediction in patients undergoing MitraClip. Clin. Res. Cardiol. Off. J. Ger. Card. Soc..

[37] Scrutinio D., Ammirati E., Passantino A., Guida P., D’Angelo L., Oliva F., Ciccone M.M., Iacoviello M., Dentamaro I., Santoro D. (2015). Predicting short-term mortality in advanced decompensated heart failure—Role of the updated acute decompensated heart failure/N-terminal pro-B-type natriuretic Peptide risk score. Circ. J. Off. J. Jpn. Circ. Soc..

[38] Codina P., Lupón J., Borrellas A., Spitaleri G., Cediel G., Domingo M., Simpson J., Levy W.C., Santiago-Vacas E., Zamora E. (2021). Head-to-head comparison of contemporary heart failure risk scores. Eur. J. Heart Fail..

[39] Lagu T., Pekow P.S., Shieh M.S., Stefan M., Pack Q.R., Kashef M.A., Atreya A.R., Valania G., Slawsky M.T., Lindenauer P.K. (2016). Validation and Comparison of Seven Mortality Prediction Models for Hospitalized Patients With Acute Decompensated Heart Failure. Circ. Heart Fail..

[40] Subramaniam A., van Houten H., Redfield M.M., Sangaralingham L.R., Savitz S.T., Glasgow A., Schulte P.J., LeMond L.M., Dunlay S.M. (2023). Advanced Heart Failure Characteristics and Outcomes in Commercially Insured U.S. Adults. JACC Heart Fail..

[41] Allen L.A., Fonarow G.C., Liang L., Schulte P.J., Masoudi F.A., Rumsfeld J.S., Ho P.M., Eapen Z.J., Hernandez A.F., Heidenreich P.A. (2009). Medication initiation burden required to comply with heart failure guideline recommendations and hospital quality measures. Circulation.

[42] Tromp J., Ouwerkerk W., Cleland J.G.F., Angermann C., Dahlstrom U., Teng K.T.H., Bamadhaj S., Ertl G., Hassanein M., Perrone S.V. (2020). Global differences in HF: The importance of region in patient characteristics, management and outcomes. Curr. Heart Fail. Rep..

[43] Canepa M., Fonseca C., Chioncel O., Laroche C., Crespo-Leiro M.G., Coats A.J.S., Mebazaa A., Piepoli M.F., Tavazzi L., Maggioni A.P. (2018). Performance of Prognostic Risk Scores in Chronic Heart Failure Patients Enrolled in the European Society of Cardiology Heart Failure Long-Term Registry. JACC Heart Fail..

[44] Khoury J., Ghersin I., Braun E., Elias A., Aronson D., Azzam Z.S., Bahouth F. (2022). Adherence to Guidelines in Heart Failure, Is It Valid for Elderly Patients?. Isr. Med. Assoc. J..

[45] Fuchida A., Suzuki S., Motoki H., Kanzaki Y., Maruyama T., Hashizume N., Kozuka A., Yahikozawa K., Kuwahara K. (2021). Prognostic significance of diastolic blood pressure in patients with heart failure with preserved ejection fraction. Heart Vessels.

[46] Bae E., Rocco M.V., Lee J., Park J.Y., Kim Y.C., Yoo K.D., Kim E.Y., Park D.J., Lim C.S., Kim Y.S. (2022). Impact of DBP on all-cause and cardiovascular mortality: Results from the National Health and Nutrition Examination survey, 1999–2014. J. Hypertens..

[47] Rodriguez M., Hernandez M., Cheungpasitporn W., Kashani K.B., Riaz I., Rangaswami J., Herzog E., Guglin M., Krittanawong C. (2019). Hyponatremia in Heart Failure: Pathogenesis and Management. Curr. Cardiol. Rev..

[48] Girerd N., Mewton N., Tartière J.M., Guijarro D., Jourdain P., Damy T., Lamblin N., Bayes-Génis A., Pellicori P., Januzzi J.L. (2022). Practical outpatient management of worsening chronic heart failure. Eur. J. Heart Fail..

[49] Task Force of the Hellenic Heart Failure Clinics Network (2020). How to develop a national heart failure clinics network: A consensus document of the Hellenic Heart Failure Association. ESC Heart Fail..

[50] McAlister F.A., Stewart S., Ferrua S., McMurray J.J. (2004). Multidisciplinary strategies for the management of heart failure patients at high risk for admission: A systematic review of randomized trials. J. Am. Coll. Cardiol..

