# Supplementary material for: Prognosis and Risk Stratification of Patients with Advanced Heart Failure Followed-Up on an Outpatient Clinic
Source: Biomedicines. 2025 Nov 10;13(11):2743. doi: 10.3390/biomedicines13112743 (PMC12650475; doi:10.3390/biomedicines13112743)
Supplement: Supplementary file 1 [file biomedicines-13-02743-s001.zip › biomedicines-3946351-supplementary.pdf]

Table 1. Subgroups analysis for factors affecting 1-year mortality

	Group 1			Group 2			Group 3		
	**OR**	**95%CI**	**P-value**	**OR**	**95%CI**	**P-value**	**OR**	**95%CI**	**P-value**
**Age/10 years increase**	1.29	0.58, 2.88	0.534	2.67	0.23, 30.62	0.429	1.16	0.40, 3.32	0.785
**Female**	0.40	0.04, 3.96	0.433	0.67	0.04, 10.25	0.771	-	-	-
**BMI per 5 kg/m2 increase**	0.69	0.29, 1.66	0.409	1.12	0.40, 3.12	0.829	0.70	0.25, 1.96	0.495
**SBP per 10 mmHg increase**	0.45	0.19, 1.04	0.061	1.61	0.74, 3.51	0.227	0.82	0.45, 1.52	0.534
**DBP per 5 mmHg increase**	0.37	0.16, 0.82	0.014	1.47	0.65, 3.33	0.357	0.61	0.30, 1.24	0.174
**PP per 10 mmHg increase**	0.98	0.46, 2.06	0.954	1.43	0.66, 3.09	0.363	1.06	0.52, 2.17	0.873
**LVEF per 10% increase**	1.06	0.47, 2.39	0.885	0.95	0.36, 2.54	0.919	0.63	0.30, 1.35	0.235
**Smoking**	3.17	0.60, 16.69	0.174	-	-	-	1.89	0.17, 21.33	0.607
**Hypertension**	0.44	0.10, 2.01	0.288	-	-	-	0.22	0.02, 2.33	0.210
**DM**	0.56	0.11, 2,72	0.469	-	-	-	1.22	0.16, 9.56	0.848
**CAD**	1.41	0.23, 8.61	0.708	6.00	0.39, 92.28	0.199	-	-	-
**COPD**	1.25	0.27, 5.77	0.775	1.00	0.07, 14.64	1.000	-	-	-
**AF**	2.13	0.36, 12.54	0.402	0.40	0.02, 6.85	0.527	-	-	-
**Hb per 1 gr/dl increase**	0.86	0.60, 1.22	0.385	1.43	0.64, 3.21	0.381	0.96	0.54, 1.71	0.899
**Na per 1 mmol/l**	0.71	0.50, 0.99	**0.047**	0.92	0.49, 1.73	0.795	0.86	0.63, 1.17	0.334

**increase**									
**K per 0.5 mmol/l increase**	0.69	0.29, 1.65	0.402	1.71	0.21, 13.99	0.618	0.67	0.22, 2.08	0.669
**eGFR per 10ml/min increase**	1.34	0.87, 2.08	0.188	0.60	0.25, 1.45	0.257	1.09	0.60, 1.99	0.778
**TNI>25 ng/ml**	1.38	0.31, 6.20	0.679	4.00	0.27, 58.56	0.311	0.33	0.03,3.50	0.360
**BNP >600 pg/ml**	0.51	0.11, 2.32	0.386	1.50	0.10, 23.07	0.771	3.18	0.30, 33.58	0.337
**Medications**									
**BB**	3.94	0.42, 37.31	0.232	0.25	0.02, 3.66	0.311	1.00	0.09, 10.74	1.000
**SGLT2i**	3.03	0.62, 14.78	0.170	0.25	0.02, 3.66	0.311	2.46	0.24, 25.67	0.453
**ACE/ARB**	1.17	0.09, 14.56	0.905	0.70	0.05, 10.01	0.793	0.45	0.04, 4.72	0.506
**MRA**	-	-	-	0.40	0.02, 6.85	0.527	-	-	-
**HCT**	0.74	0.07, 8.13	0.806	-	-	-	-	-	-
**Furosemide dose >120 mg**	0.44	0.10, 2.01	0.288	0.17	0.01, 2.56	0.199	12.00	1.10,131.24	**0.042**

ACE/ARB= angiotensin-converting enzyme inhibitors/ angiotensin II receptor blockers, AF= atrial fibrillation, BB=beta blockers, BMI= body mass index, BNP= brain natriuretic peptide, CAD= coronary artery disease, COPD= chronic obstructive pulmonary disease, CRTD= cardiac resynchronization therapy defibrillator, CRTP= cardiac resynchronization therapy pacemaker, DBP=diastolic blood pressure, DM=diabetes mellitus, eGFR= estimated glomerular filtration rate, HB= hemoglobin, HCT= hydroclorothiazide, ICD= implantable cardioverter defibrillator, LVEF=left ventricle ejection fraction, MRA= mineralocorticoid receptor antagonist, PCM=pacemaker, PP= pulse pressure, SBP= systolic blood pressure, SGLT2I= Sodium-Glucose contransporter 2 inhibitors, TNI= troponin-I

Table 2. Subgroup analysis for factors affecting 30 months mortality

	Group 1			Group 2			Group 3		
	**OR**	**95% CI**	**Pvalue**	**OR**	**95% CI**	**Pvalue**	**OR**	**95% CI**	**Pvalue**
**Age/10 years**	1.42	0.65, 3.11	0.386	-	-	-	0.90	0.42, 1.94	0.795

**increase**									
**Female**	0.32	0.04, 2.75	0.297	-	-	-	0.45	0.08, 2.54	0.366
**BMI per 5 kg/m2 increase**	0.67	0.27, 1.64	0.376	-	-	-	0.99	0.58, 1.68	0.957
**SBP per 10 mmHg increase**	0.63	0.36, 1.08	0.092	-	-	-	0.67	0.41, 1.10	0.114
**DBP per 5 mmHg increase**	0.64	0.41, 1.01	0.057	-	-	-	0.58	0.35, 0.98	**0.041**
**PP per 10 mmHg increase**	0.85	0.39, 1.86	0.684	-	-	-	0.76	0.43, 1.36	0.352
**LVEF per 10% increase**	0.88	0.36, 2.11	0.770	-	-	-	0.54	0.32, 0.92	**0.024**
**Smoking**	1.20	0.19, 7.70	0.848	-	-	-	0.41	0.04, 3.88	0.435
**Hypertension**	0.67	0.11, 4.20	0.666	-	-	-	1.13	0.26, 4.89	0.875
**Diabetes**	2.73	0.44, 16.75	0.279	-	-	-	2.00	0.46, 8.70	0.355
**CAD**	2.00	0.27, 14.98	0.500	-	-	-	2.69	0.58, 12.60	0.208
**COPD**	1.25	0.24, 6.65	0.794	-	-	-	2.00	0.46, 8.70	0.355
**AF**	3.20	0.58, 17.72	0.183	-	-	-	3.60	0.39, 33.24	0.259
**Hb per 1 gr/dl increase**	0.77	0.51, 1.18	0.237	-	-	-	0.95	0.61, 1.47	0.805
**Na per 1 mmol/l increase**	0.61	0.38, 0.98	**0.04**	-	-	-	0.77	0.60, 0.98	**0.034**
**K per 0.5 mmol/l increase**	1.50	0.54, 4.17	0.441	-	-	-	0.80	0.38, 1.68	0.561
**eGFR per 10ml/min increase**	0.91	0.60, 1.40	0.671	-	-	-	1.18	0.74, 1.87	0.495
**Tni >25 ng/ml**	6.00	0.95, 37.76	0.056	-	-	-	0.62	0.14, 2.70	0.523
**BNP >600 pg/ml**	1.83	0.35, 9.72	0.476	-	-	-	2.15	0.44, 10.44	0.341
**Medications**<br>**BB**	3.60	0.55, 23.65	0.182				0.33	0.07, 1.60	0.168

**SGLT2i**	11.38	1.17, 110.42	0.036				2.33	0.50, 10.91	0.282
**ACE/ARB**	0.74	0.06, 9.46	0.815				0.50	0.11, 2.31	0.371
**MRA**	-	-	-				-	-	-
**HCT**	-	-	-	-	-	-	-	-	-
**Furosemide dose >120 mg**	1.63	0.32, 8.40	0.562	-	-	-	3.07	0.62, 15.08	**0.168**

ACE/ARB= angiotensin-converting enzyme inhibitors/ angiotensin II receptor blockers, AF= atrial fibrillation, BB=beta blockers, BMI= body mass index, BNP= brain natriuretic peptide, CAD= coronary artery disease, COPD= chronic obstructive pulmonary disease, CRTD= cardiac resynchronization therapy defibrillator, CRTP= cardiac resynchronization therapy pacemaker, DBP=diastolic blood pressure, DM=diabetes mellitus, eGFR= estimated glomerular filtration rate, HB= hemoglobin, HCT= hydroclorothiazide, ICD= implantable cardioverter defibrillator, LVEF=left ventricle ejection fraction, MRA= mineralocorticoid receptor antagonist, PCM=pacemaker, PP= pulse pressure, SBP= systolic blood pressure, SGLT2I= Sodium-Glucose contransporter 2 inhibitors, TNI= troponin-I
